# A Novel Alignment-Free Method for Comparing Transcription Factor Binding Site Motifs

**DOI:** 10.1371/journal.pone.0008797

**Published:** 2010-01-20

**Authors:** Minli Xu, Zhengchang Su

**Affiliations:** Department of Bioinformatics and Genomics, University of North Carolina at Charlotte, Charlotte, North Carolina, United States of America; Dana-Farber Cancer Institute, United States of America

## Abstract

**Background:**

Transcription factor binding site (TFBS) motifs can be accurately represented by position frequency matrices (PFM) or other equivalent forms. We often need to compare TFBS motifs using their PFMs in order to search for similar motifs in a motif database, or cluster motifs according to their binding preference. The majority of current methods for motif comparison involve a similarity metric for column-to-column comparison and a method to find the optimal position alignment between the two compared motifs. In some applications, alignment-free methods might be preferred; however, few such methods with high accuracy have been described.

**Methodology/Principal Findings:**

Here we describe a novel alignment-free method for quantifying the similarity of motifs using their PFMs by converting PFMs into *k*-mer vectors. The motifs could then be compared by measuring the similarity among their corresponding *k*-mer vectors.

**Conclusions/Significance:**

We demonstrate that our method in general achieves similar performance or outperforms the existing methods for clustering motifs according to their binding preference and identifying similar motifs of transcription factors of the same family.

## Introduction

Transcription factors (TFs) play important roles in the regulation of gene transcription through binding to specific DNA sequences called TF binding sites (TFBSs), which are usually 5–25 bp in length [1], [2]. The TFBSs of the same TF show some level of conservation but are rather degenerate, and they are collectively called a TFBS motif in this paper. A TFBS motif is often represented by a position frequency matrix (PFM), which consists of nucleotide frequencies at each position of the motif [3]. A PFM is derived from the alignment of known TFBSs of the TF, and it largely reflects the TF's DNA binding preference at each position. Thus once the PFM of a TF is known, it is possible to predict other binding sites by scanning it against the regulatory regions in a genome [4], [5].

It is often desired to compare the similarity among motifs using their PFMs to either infer the cognate TF of a putative motif by comparing it with known motifs in a database or to cluster redundant or sub-motifs of the same TF or motifs of related TFs. For instance, it has been suggested that PFMs of different TFs from a structurally related class can be merged to form a generalized binding model or familial binding profile (FBP) [6]. An FBP model reflects the “average” binding preference of the TFs in the family and can be incorporated in motif finding algorithms as prior knowledge to increase the sensitivity in finding motifs for a particular TF family [6]–[8]. Furthermore, in genome-scale TFBS prediction applications, redundant and sub motifs of the same TFs are often returned by motif finders, and they need to be clustered to form unique motifs [9], [10]. In all these applications, the similarity between two motifs needs to be accurately calculated for the desired purposes.

Current methods for motif comparison typically involve a similarity metric for column-to-column comparison and a method to find the optimal position alignment between the two compared motifs [8]. The final similarity score between the two motifs is computed based on the alignment of columns and the chosen column similarity metric. The column similarity metrics that have been used include Pearson's correlation coefficient (PCC), *p*-value of Chi-square (pCS), average Kullback-Leibler (KL), Sum of squared distances (SSD), and average log likelihood ratio (ALLR), etc. [6], [9], [11]–[15]. Either a global or local optimal column-to-column alignment between two PFMs is typically generated using dynamic programming, such as the Smith-Waterman [16] or Needleman-Wunsch algorithm [17]. The combinations of these column similarity metrics and alignment methods have been thoroughly evaluated recently by Mahony and Benos [8], and implemented in the software package STAMP [7]. More recently, an alignment free motif comparison method MoSta was proposed by Pape *et al.* [18].

In this paper we present a new alignment-free method for motif comparison, which was largely inspired by the strategies for alignment-free sequence comparisons [19]. Briefly, we first convert each PFM into a composition vector with each element representing the likelihood score for a particular short *k*-mer sequence fitting the PFM model. Therefore, the vector, which we term a *k*-mer frequency vector (KFV), contains scores for all possible *k*-mer words. We then compute the similarity between two motifs using a distance measure between their corresponding KFVs. In this way we eliminate the necessity of the alignment step while effectively capturing the similarity between the two compared motifs.

In the following sections, we will first describe our algorithm in detail, and then present its performance for motif retrieval and clustering compared with other state-of-the-art methods.

## Methods

### 1. Datasets of PFMs

A TFBS motif of length *n* (bp) is usually represented as a 4×*n* matrix in a variety of forms such as position frequency matrix (PFM), position weight matrix (PWM), position specific scoring matrix (PSSM/PSM), etc. These matrices reflect various features of the sequence motifs including frequency of occurrence, probability, log-likelihood, etc. Although some form may be a better representation of sequence motifs for a certain purpose, they all convey similar information about motifs. Therefore in this study we focus on the comparison of PFMs, as the other matrix representations can be easily derived from the PFMs. In this regard, we designed our algorithm to take PFMs as the input. Specifically, we define a PFM as the nucleotide frequency at each position from the aligned motif sequences. Three datasets of experimentally verified PFMs were used for testing and evaluation purpose in this study (Table 1). Dataset-1 and Dataset-2 contain PFMs with known TF structural classes in JASPAR [20], [21] and TRANSFAC [22] respectively. Dataset-1 was also used by Mahony *et al.* [8], containing 96 PFMs from JASPAR, and 25 of them are from Zinc-Finger (ZF) families. Dataset-2, created by Narkilar *et al.* [23], contains 355 PFMs from six large TF structural families in TRANSFAC. To compare our algorithm to the existing methods for their ability to detect redundant PFMs without considering structure similarity, we created Dataset-3 based on the 124 JASPAR core motifs downloaded from JASPAR (http://jaspar.genereg.net/html/DOWNLOAD/SITES/JASPAR_CORE_2008/). We first created the PFM for each of the 124 binding site alignments. We then created additional three PFMs for each alignment by randomly removing (without replacement) one-third of the sequences from the motif alignment. This leads to total 124*4 = 496 PFMs in Dataset-3. The basic information about the three datasets is summarized in Table 1.

**Table 1:** Three datasets used in this study for testing and evaluation.

Dataset	Dataset-1	Dataset 2	Dataset-3
Number of PFMs	96	355	496
Average length	10.39	12.14	10.6
Min length	4	4	4
Max length	30	29	22
Number of Classes	13	6	-
PFM sources	JASPAR	TRANSFAC	JASPAR
Dataset source	Mahony, et al., 2007	Narlikar and Hartemink, 2006	This study

### 2. Conversion of a PFM into a KFV

Let PFM *M* be a 4×*n* matrix with each column being the frequencies of the four types of nucleotides at that position in the alignments of the TFBSs with length *n*. A sequence of *k* (*k*≤*n*) nucleotides is designated as a *k*-mer (*K*). Let *S_k_* be the set containing all possible *k*-mers. Clearly, *S_k_* has 4*^k^* elements:
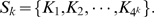



We construct a 4*^k^*-dimensional KFV *V_M_* to represent *M*, with each element in *V_M_* being the likelihood (*L*_*Ki,M*_) for a specific *k*-mer (*K* *_i_*) being described by *M*,
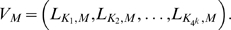



Intuitively, to compute the likelihood *L*_K,*M*_, we slide the *k*-mer *K* over the motif PFM *M*, and for each shift, we calculate a probability that *K* fits the corresponding columns of *M*. We then sum up this probability scores over all shifts as the likelihood of *K* fitting *M*. Formally, *L*_K,*M*_ is defined as,
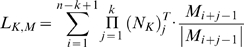



In the above equation, *n* is the length of *M*, *k* is the length of the *k*-mer and *k≤n*, and *N_K_* is the 4×*n* (bits) matrix form of the *k*-mer (*K*), with “1” in a column representing certain nucleotide at that position in the *k*-mer. For instance, the 5-mer TAGAC can be presented by the following 4×5 matrix:
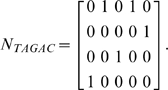
(*N_K_*)_j_ is the *j*-th column of *N_K_*, *M_i_* is the *i*-th column of *M* and | *M_i_* | is the Manhattan norm of column vector *M_i_*, defined as:
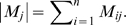



### 3. Comparison between Two KFVs

After PFMs are converted into KFVs, a distance *d*(*a,b*) between two KFVs *a* and *b* can be defined such that it possesses the following three properties according to Strang's definition of distance metric [24]:
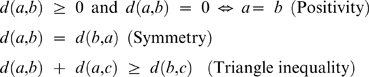



Clearly, many distance/dissimilarity metrics defined between two vectors meet the above criteria, and thus can be used to measure *d*(*a,b*). These metrics include Euclidean distance [25], *d2* distance [26], Pearson correlation coefficient (PCC) [27], [28], Mahalanobis distance [29], information theory based measure using Kullback-Leibler (KL) discrepancy [30], angle metrics [31], [32], etc.. For a review of these methods see [19]. Here we evaluated our method on four distance metrics including the Euclidean distance (d_Euclidean_), Pearson correlation (d_PCC_), cosine angle metric (d_cos_), and a modified KL distance (d_KL_) for measuring the dissimilarity between KFVs. Specifically, for KFV *a* and *b*, these distance metrics are defined respectively as below:
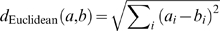


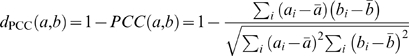


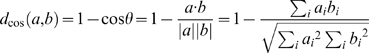


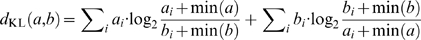
where *a_i_* and *b_i_* are the *i*-th element of *a* and *b* respectively, and 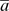 and 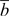 are average of elements of *a* and *b*, respectively. Both PCC and cosine angle metric are scale-independent, so that they are not sensitive to repetitions, which often occur in TFBS motifs such as tandem repeats and palindromic structures. The KL discrepancy is an information theory-based metric, measuring relative entropy between two discrete probability distributions. The modified KL distance (*d_KL_*) is actually a sum of two KL discrepancy values, so that the *d_KL_* distance can be symmetric. To avoid zero on the denominator, a pseudo-number, which is the minimum of *a* or *b*, was added to both *a_i_* and *b_i_*. Both *d_PCC_* and *d_cos_* have the range of [0,1] while *d_KL_* and *d_Euclidean_* range from 0 to +∞.

In practice, we may not know the orientation of motifs compared, thus we compute two distance scores for each pair of PFMs for the two possible orientations. We then take the smaller one as the distance between the two PFMs:
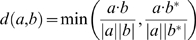
where b* is the KFV derived from the reverse complement of the same PFM (*b*).

### 4. Performance Evaluation by ROC Analysis

We employed the ROC analysis to compare our algorithm to other prior methods for their ability to identify the TFBS motifs of structural and/or evolutionarily related TFs in the above mentioned three datasets, for Dataset-1 and Dataset-2, the ROC curves were plotted based on the following criteria. Given a dataset containing *N* PFMs with known TF structural classes, N(N+1)/2 pair-wise comparisons (including self-comparisons) were conducted and pair-wise similarity scores were computed using our algorithm or the other compared methods. We consider a pair of PFMs as a match (positive) if the distance *d*(*a,b*) between their corresponding KFVs *a* and *b* is within a threshold, or a mismatch (negative), otherwise. We consider a positive as a true positive if the two associated TFs come from the same structural class, and a negative as a true negative if the associated two TFs are from different structural classes. The ROC curve plots the true positive rate (TPR) against the false positive rate (FPR), computed for different thresholds of pair-wise distances.

For Dataset-3, the ROC curves were plotted using the similar criteria as described above, except that we consider a positive as a true positive if the two PFMs are originated from the same motif alignment, and a negative as a true negative if the two PFMs are originated from the different motif alignment. This assignment of true positives/negatives could allow us to compare motif metrics for their ability to detect redundant PFMs in a set of motifs.

### 5. Hierarchical Clustering and Motif Tree Construction

To test if our motif comparison algorithm is effective for clustering similar motifs, we computed pair-wise distances for the 71 non-ZF motifs in Dataset-1, which was used previously by Sandelin and Wasserman [6] to construct familial binding profiles (FBP) and more recently by Mahony *et al.* [8] for PFMs clustering. The pair-wise distances were computed using a word size *k* = 4, since this *k* value generally gives the best performance based on our experiments (see section 3.1 and also Figure S1). To better display the resulting PFM trees, we transform the above defined distance using the following exponential function,
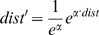
where α is a constant, and we chosen α = 10 in this application. Finally, the UPGMA algorithm was used to hierarchically cluster these 71 non-ZF PFMs.

### 6. Estimation of the Optimal Number of Clusters and Construction of FPMs

We used the statistic *CH_log_* to estimate the optimal number of clusters from the UPGMA clustering result. *CH_log_* proposed by Mahony *et al.* [8], is a derivative of the *CH* index [33] aiming to find an optimal balance between inter-cluster and intra-cluster variability, and is defined as,
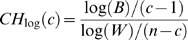
where *B* and *W* represent the sum of between (inter-) and within (intra-) cluster distances, respectively, and *n* and *c* are the number of data points (PFMs) and number of clusters under consideration, respectively. The optimal number of clusters is indicated by the *c* value that maximizes *CH_log_*.

Based on the estimated optimal number of clusters, a FBP was generated for each cluster using the STAMP package [7].

### 7. Database Searching and Implementation

We have implemented the algorithm as a web server for demonstration purpose, with which a user can identify the best matching motifs in a specified motif database of a query motif. The server can be accessed at http://bioinfo.uncc.edu/kfv/

## Results

### 1.Performance of the KFV Algorithm for Motif Retrieval with Various Parameter Settings

It has been shown that structurally and/or evolutionarily related TFs tend to bind similar TFBS motifs, however, identification of these similar motifs can be difficult due to the highly degenerate nature of TFBSs. We therefore evaluated our algorithm for identifying the TFBS motifs of structural and/or evolutionarily related TFs using all three datasets by the “best-hit” approach used in Mahony *et al.* [8]. Specifically, for Dataset 1 and 2, we asked if the TF of the best match of the returned hits shared the same structural class of the TF of the query PFM, when the PFM was queried against a dataset containing multiple PFMs. For Dataset-3, we checked whether the ‘best-hit’ was originated from the same motif alignment as the query PFM. Following the practice of Mahony *et al.* [8], we define the accuracy of a method as the percentage of motifs whose structural classes are correctly recovered by the method as the best-hit in the database searches. The performance of KFV on all three datasets at various parameter settings are listed in Table 2.

**Table 2:** Performance of the KFV algorithm on the three datasets measured as the accuracy from the “best hit” test.

Dataset-1	k = 1	k = 2	k = 3	k = 4	k = 5
- Euclidean	0.385	0.708	0.760	0.792	0.674
- PCC	0.250	0.677	0.802	0.823	0.768
- cosine angle	0.375	0.719	0.823	**0.833**	0.768
- KL-based	0.427	0.646	0.729	0.667	0.568
Dataset-2	k = 1	k = 2	k = 3	k = 4	k = 5
- Euclidean	0.531	0.789	0.854	0.868	0.859
- PCC	0.251	0.803	0.882	0.899	0.898
- cosine angle	0.475	0.800	0.873	**0.901**	0.893
- KL-based	0.562	0.777	0.811	0.823	0.805
Dataset-3	k = 1	k = 2	k = 3	k = 4	k = 5
- Euclidean	0.946	**0.988**	0.984	0.986	0.986
- PCC	0.323	0.978	0.980	0.984	0.984
- cosine angle	0.760	0.982	0.982	0.984	0.986
- KL-based	0.921	0.974	0.972	0.964	0.953

For Dataset-1 and 2, as expected, when *k* = 1 the accuracy of the KFV algorithm was low (from 0.25 to 0.56), since at this k value the algorithm was reduced to a method comparing motifs based solely on the nucleotide composition of PFMs. Starting from *k* = 2, our algorithm began to gain discriminative ability and reached the maximal overall accuracy when *k* = 4 for both dataset-1 and 2 using all four vector distance metrics with the exception that the KL-based distance has the optimal *k* = 3 for Dataset-1. These results suggested that in general PCC and cosine angle distance had better performance than Euclidean distance and the KL-based distance. It is not very surprising that PCC and cosine angle distance have similar performance, as both metrics are mathematically similar and related (see section 2.3). Since the combination of cosine angle metric and k = 4 gave the highest overall accuracy, we used k = 4 and cosine angle as the parameter settings in the rest of the experiments in this study. For Dataset-3, since the PFMs are originated from the same motif alignment, their differences are small, good accuracy could be achieved even for *k* = 1. Although the highest accuracy (0.988) was achieved when *k* = 2 and Euclidean distance was used, we still used the combination of cosine distance and k = 4 in later analysis on this dataset since this combination also has very good accuracy (0.984).

### 2.Comparison of the KFV Algorithm with Other Methods for Motif Retrieval

We first compared our method to the six well-regarded alignment-based methods implemented in the STAMP package, which represents state of the art research of motif comparision [8] and a more recently developed alignment-free method MoSta [18], for their ability to identify the TFBS motifs of structurally and/or evolutionarily related TFs using Dataset-1 by the “best-hit” method. The methods in STAMP can be selected by specifying the column comparison metric and alignment method [7]. MoSta uses asymptotic covariance to measure the natural similarity between two PFMs without the requirement of alignment [18].

As shown in Table 3, our KFV method with *k* = 4 and cosine angle outperforms all the six major methods implemented in STAMP on the 71 non-ZF PFMs, and achieves the same high retrieval accuracy of 0.915 as MoSta (S_max_). On the other hand, the KFV method outperforms MoSta using either of its similarity measures on the 25 ZF PFMs, and achieves the same high retrieval accuracy of 0.6 as STAMP using PCC as the column similarity metric. The accuracy of our algorithm as well as that of the methods in STAMP and MoSta for the ZF PFMs is lower than that achieved for the non-ZF PFMs. This might be largely due to the fact that among the 25 ZF PFMs, 17 are from the ZF-C2H2 family containing TF proteins with highly divergent binding motifs. Nevertheless, our method achieves an accuracy of 0.833 (the fourth column in Table 3) on the entire Dataset-1, which is higher than any method in STAMP or MoSta.

**Table 3:** Comparison of the KFV algorithm with other methods for motif retrieval using Dataset-1.

	Accuracy		
Method	Non-ZF PFMs(71)	ZF PFMs (25)	Total (96)
KFV (k = 4, cosine)	**0.915**	**0.600**	**0.833**
STAMP (PCC)	0.887	**0.600**	0.813
STAMP (SSD)	0.859	0.560	0.781
STAMP (AKL)	0.831	0.520	0.750
STAMP (ALLR-LL)	0.859	0.400	0.740
STAMP (pCS)	0.761	0.560	0.708
STAMP (ALLR)	0.775	0.400	0.677
MOSTA (S_max_)	**0.915**	0.440	0.792
MOSTA (S_sum_)	0.817	0.560	0.750

The results are shown separately for the zinc-finger and non zinc-finger families. The values in bold indicate the highest accuracy achieved for each category. In parentheses beside each method are the primary parameter settings (column comparison metric for STAMP or similarity measure score for MoSta). The accuracy for STAMP using different column comparison metrics were taken from [8], in which the evaluation was performed using the optimal alignment strategies and gap scores on the same dataset. For MoSta, a GC content of 0.5 and the balanced threshold were used.

We then compared the performance of our algorithm for motif retrieval on Dataset-2 by the ‘best–hit’ method to that of STAMP [8] and a Bayesian learning algorithm [23] as both algorithms have been applied by their authors to this dataset for the same purpose. We also included MoSta in this test (a GC content of 0.5 and the balanced threshold were used). As shown in Table 4, our KFV algorithm (*k* = 4 and cosine angle) outperforms both STAMP and Bayesian Learning on all these TF families with the exception for the bZIP family where our algorithm achieves similar accuracy to the Bayesian Learning method but is worse than STAMP. Compared with MoSta, both KFV and MoSta have similar performance on this dataset except that KFV performs better in C2H2 family. In general, our KFV algorithm can achieve similar performance to these current existing methods, and it has slightly higher overall performance (see the last row in Table 4).

**Table 4:** Comparison of the KFV algorithm with other methods for motif retrieval using Dataset-2.

Structural Class	Accuracy			
	KFV	STAMP	MoSta (Smax/Ssum)	Bayesian Learning
bZIP (93)	0.92	**0.94**	0.90/**0.94**	0.92
C2H2 (74)	**0.82**	0.76	0.76/0.72	0.77
C4 (52)	**0.98**	**0.98**	**0.98**/0.94	0.91
Homeo (50)	0.88	0.82	0.82/**0.92**	0.85
Forkhead (49)	**0.92**	0.9	**0.92**/0.86	0.83
bHLH (37)	0.89	0.81	**0.92**/0.73	0.88
*Total (355)*	***0.90***	*0.87*	*0.88/0.86*	*0.86*

The number in the parentheses is the number of PFMs within that TF structural class. The accuracies for STAMP and Bayesian Learning were taken from Mahony *et al.* [8]. The accuracy for STAMP was evaluated using ungapped Smith-Waterman alignment and PCC metric for column comparison. The accuracy for KFV was evaluated with *k* = 4 and cosine angle distance. The values in bold font indicate the highest accuracy achieved for each structural class.

**Figure 1:**
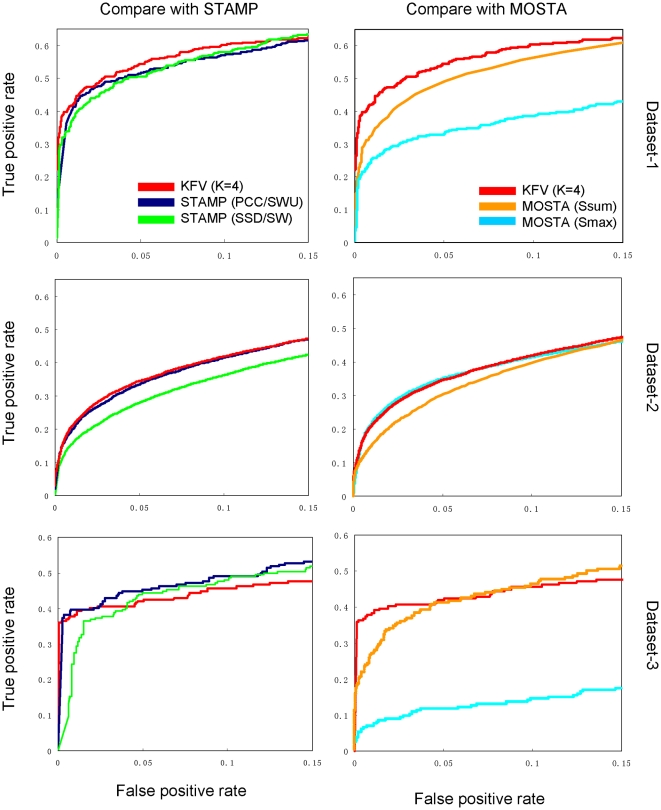
Evaluation of three motif comparison algorithms using ROC curves. The ROC curves were plotted based on three datasets in Table 1.

To further compare our algorithm with STAMP and MoSta, we conducted ROC analysis of the performance of the three packages on the three datasets outlined in section 2.1. Notably, we run STAMP [7], [8] using PCC with ungapped Smith-Waterman alignment (PCC/SWU) and SSD with gap open = 1 and gap extension = 0.5 (SSD/SW) as the algorithms because they have the best overall performance among others according to the authors. For MoSta, we used a GC content of 0.5 (50%) and the balanced threshold [18]. For each pair of PFMs, MoSta returns two similarity scores (S_max_ and S_sum_). As shown in Figure 1, our algorithm clearly outperforms both algorithms in STAMP (PCC/SWU and SSD/SW) as well as both metrics in MoSta on Dataset-1, which is consistent with the results shown in Table 3. The performance of our algorithm on Dataset-2, is also better than both algorithms in STAMP, while MoSta (S_max_ score) slightly outperforms our algorithm when the false positive rate is below 0.07. Although all the three methods perform well on Dataset-3 (Figure 1) because the PFMs derived from the same motif alignment are generally very similar to each other, our KFV method clearly outperforms both STAMP and MoSta. This indicates that KFV could be a very useful metric for detecting highly similar redundant PFMs in a motif dataset. These ROC analyses on the three datasets again suggest that our method is rather accurate, and the parameters setting (k = 4 and cosine angle metric) is very robust in identifying similar and redundant motifs in a motif dataset, and thus could be used for general purpose.

### 3.Performance of the KFV Algorithm for Hierarchical Motif Clustering

**Figure 2:**
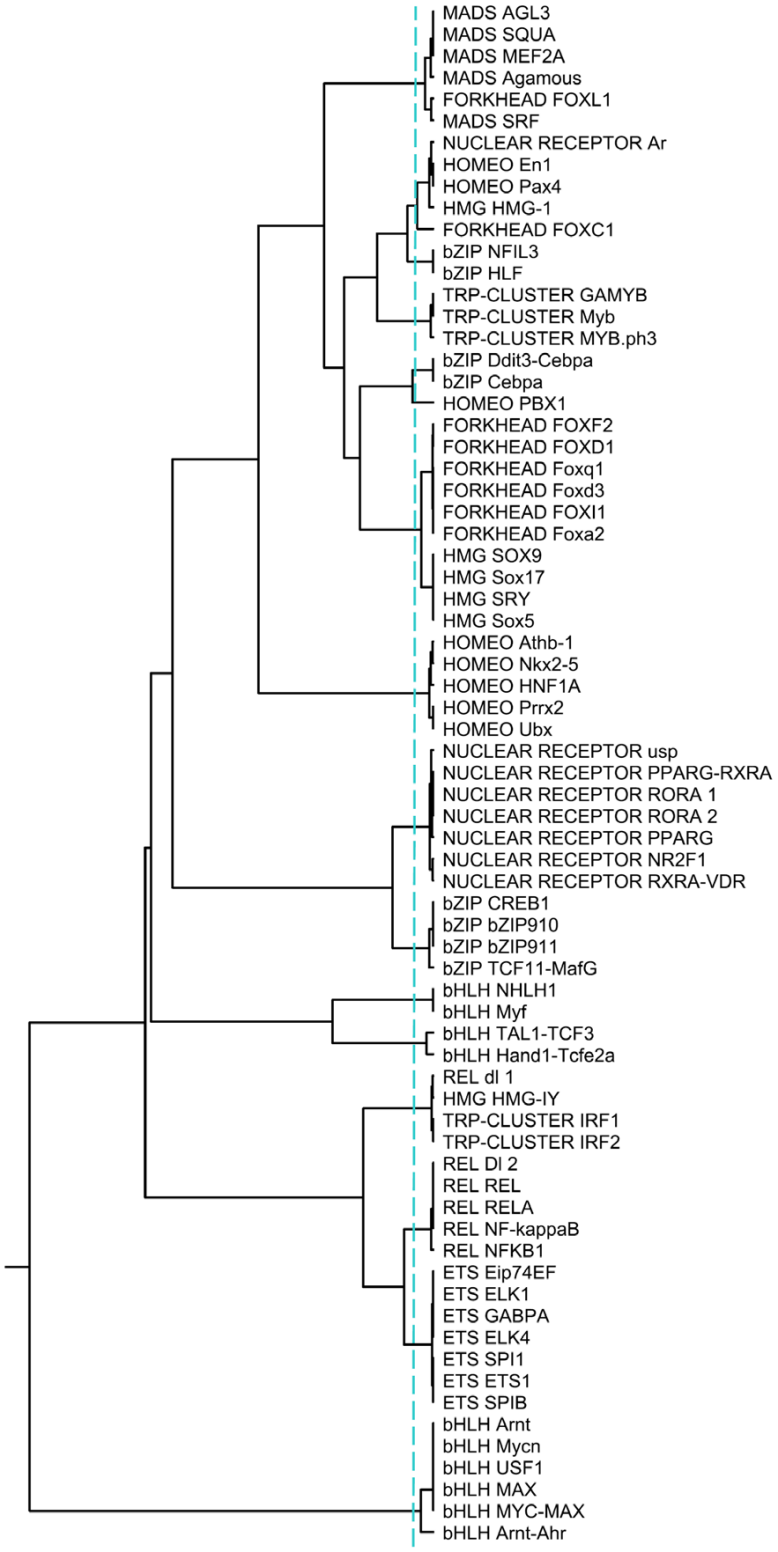
The motif tree of the 71 non-ZF PFMs in Dataset-1. The tree was constructed using the UPGMA algorithm based on the pairwise distances calculated by our KFV method with *k* = 4 and cosine angle metric. The vertical dashed blue line represents the level at which the *CH_log_* metric estimates the optimal number of clusters. The 71 PFMs were grouped into 16 groups as indicated by the dashed line.

**Figure 3:**
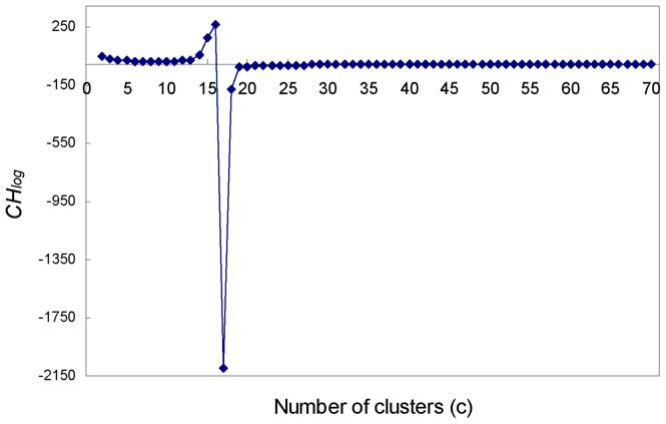
The *CH_log_* plot. The log modified Calinski and Harabasz metric (*CH_log_*) at different hierarchical levels (from 2 to 70 clusters) for the motif tree in Figure 2. The maximal *CH_log_* value was reached when the number of clusters (*c*) was 16, suggesting an optimal number of 16 groups of PFMs for the 71 non-ZF PFMs in Dataset-1.

Lastly, we evaluated our algorithm for hierarchical clustering of similar motifs. To this end, we constructed a motif tree (PFM tree) of the 71 non-ZF JASPAR PFMs from Dataset-1 using our KFV algorithm with k = 4 and cosine angle being the distance metric (Figure 2). The intra/inter cluster stability analysis of this tree using the statistic *CH_log_* suggests an optimal number of clusters of 16 for the 71 PFMs with one singleton cluster containing a PFM HOMEO PBX1 (Figure 3). The logos of the remaining 15 clusters, each is represented as a FBP, are shown in Figure S2. As shown in Figure 2, overall, the dataset was grouped into homogeneous clusters with respect to the structural classes of the corresponding TFs. This result is consistent with the commonly accepted notion that structurally related TFs may have similar binding preference to DNA sequences, since the hierarchical clustering performed was solely based on the binding preference information (PFMs).

In general, our clustering result on this dataset was quite similar, albeit with subtle difference, to the result from Mahony *et al.* [8] on the same dataset. Among the 71 PFMs, all seven from the ETS family were clustered together and formed a monophyletic group. The NUCLEAR RECEPTOR, HOMEO and REL clusters were homogeneous, although some members of these families appear in other clusters. The PFMs of the bHLH family were clustered into three homogeneous groups, with six PFMs forming a bHLH-zip subclass in one group and four PFMs in other two separate but close clusters. The bZIP motifs were also clustered into three separate groups, with four PFMs (CREB-like) in one cluster and four (C/EBP-like) in the other two separate clusters. It should be noted that one PFM from the FORKHEAD family (FOXL1) was clustered with five MADS motifs, which could be explained by the fact that TFBSs for FOXL1 contains only 3 information-rich positions, which resembles partial MADS TFBSs. As shown in Figure 2, the PFMs of the FORKHEAD and HMG families could not be separated from each other. This was largely because the binding sites of both TF families contain an AT rich core and there was an overlap in four positions in these binding sites with high information content.

Although our motif tree is in general very similar to that of Mahony *et al.* (see Figure 5 in [8]), our tree seems to make more biological sense. For instance, although both STAMP and our algorithm clustered the 10 bHLH PFMs into three clusters, our algorithm grouped the two clusters containing “standard” (non-bHLH zip) bHLH PFMs closer to each other, while STAMP grouped one cluster containing Myf and NHLH1 close to the bHLH-zip cluster. On the other hand, both STAMP and our algorithm failed to separate FPMs of the HMG and FORKHEAD families, and grouped them together in a single cluster. However, as shown in Figure 2, our algorithm grouped FORKHEAD and HMG PFMs into two homogenous subgroups within that cluster, while STAMP mixed the HMG subgroup with a HOMEO PFM. It should be also noted that STAMP could separate the FOXL1 cluster from the MADS cluster and assign FOXL1 a singleton cluster so that MADS formed a homogenous group, while our algorithm failed to do so, generating a non-homogenous MADS cluster.

We also constructed a motif tree using the MoSta method (S_max_). The tree (Figure S3) is quite similar to the one constructed using the KFV algorithm (Figure 2). This result is expected as both algorithms achieved the same accuracy for motif retrieval on those 71 non-ZF PFMs from Dataset-1 (Tble-3).

## Discussion

Compared with the existing well-regarded alignment-based motif similarity comparison methods (e.g. the methods in STAMP), our method do not require an alignment between the two compared motifs, thus it is free from the influence of the largely arbitrary choice of the multiple parameters used in an alignment method. Although sometime it is desired to have different parameters for different applications, the real-valued gap opening and extension scores as well as the choice of alignment methods make it hard to optimize these parameter settings for a certain task. Although our KFV approach is not parameter-free, it only contains two parameters, that is, the *k*-mer length *k* and the vector distance metric. Moreover, the constraint on the choice of *k* from the motif length (5–25 bp) [1], [2] and its integer-valued nature make *k* be easily optimized for a vector distance metric. Intuitively, the choice of *k* value depends on the distribution of the length of motifs in a dataset. As indicated in the Figure S4, the distributions of the length of motifs in the three datasets are very similar with an average length about 10 bp, this might explain why our KFV algorithm performs equally well in the three datasets with the same *k* value (*k* = 4). Because a motif length is determined by biophysical principles of protein-DNA interactions, its distribution should be universal, rather than dataset- or genome-dependent as long as the dataset is well-sampled. Thus we believe that our choice of *k* = 4 should be rather robust for the KFV algorithm. Furthermore, we showed that our algorithm performs well with cosine angle as the vector distance measure on the all the three datasets, suggesting the combination of *k* = 4 and cosine angle could be used as default parameters for general purpose. More importantly, our algorithm achieved at least similar accuracy to the best-regarded methods in STAMP (Table 3, and 4). Of course, one distinct advantage of alignment-based methods is their faster running time, especially for ungapped alignment (see Table S1). When ungapped alignment strategy is chosen, the alignment task is reduced to a much simpler position shift problem, and Mahony *et al.* found that ungapped alignment works well for most datasets [8].

In addition, our algorithm achieves similar performance and outperforms the recently developed alignment-free motif comparison method, MoSta [18] in most of the cases tested (Table 3, 4 and Figure 1). Our algorithm is also much faster than MoSta (Table S1). Although both algorithms are based on the word statistics of motifs, they differ significantly from each other in algorithmic designs. Specifically, MoSta measures the ‘natural similarity’ between two PFMs by calculating the asymptotic covariance between the sets of compatible words associated with the two PFMs compared. Although the idea of compatible words is similar to that of *k*-mers in our algorithm, compatible words need to be full-length binding site sequences, while *k*-mers have a fixed length and are usually short (e.g. *k* = 4). In order to construct a compatible word set, a threshold value is needed to filter out ‘non-compatibles’ and only compatible words for each PFM are kept. On the other hand, KFV keeps all the *k*-mers and their likelihood scores, which are used for the comparison between PFMs. While enumerating all possible *k*-mer (e.g. 256 for 4-mer) is fast, searching through the sequence space to construct a compatible words set is time consuming, especially for TFBSs (PFMs) with relatively long lengths, explaining why MoSta is much slower than KFV (Table S1).

To conclude, our method in most cases can achieve similar performance to or sometimes outperform other state-of-the-art methods for identifying similar or redundant motifs in a database as well as for clustering similar motifs of structurally or evolutionarily related TFs. In this sense, it can be at least used as an alternative to the current motif comparison methods. In particular, we have shown that our method can be a better choice for motif retrieval from a database and identifying highly similar redundant motifs in a motif dataset. Additionally, due to its robustness, our algorithm can be used in a wide range of applications. In particular, as there are more and more studies focusing on transcription regulation in both prokaryotes and eukaryotes, TFBS data will increase exponentially in the next several years. Therefore, it is foreseeable that more motif databases will be created. We hope that our algorithm could contribute to the efficient utilization of these databases.

## Supporting Information

Figure S1 The ROC curves for KFV with different k values ranging from 1 to 5.
(0.21 MB DOC)

Figure S2 The logos of 15 familial binding profiles (FBP) based on the 16 clusters from the motif tree (Figure 2).
(0.21 MB DOC)

Figure S3 The motif tree of the 71 non-ZF PFMs in Dataset-1 constructed using MoSta.
(0.04 MB DOC)

Figure S4 Distributions of motif length in the three datasets.
(0.03 MB DOC)

Table S1 Comparison of the running times of KFV, STAMP, and MoSta.
(0.03 MB DOC)
