# Supplementary material for: A Novel Alignment-Free Method for Comparing Transcription Factor Binding Site Motifs
Source: PLoS One. 2010 Jan 20;5(1):e8797. doi: 10.1371/journal.pone.0008797 (PMC2808352; doi:10.1371/journal.pone.0008797)
Supplement: Figure S1 — The ROC curves for KFV with different k values ranging from 1 to 5. (0.21 MB DOC) [file pone.0008797.s001.doc]

**Figure S1**. **The ROC curves for KFV with different *k* values ranging from 1 to 5.** The criteria to generate these ROC curves are the same as those generated in the main text (Section 2.4) and the datasets used here are Dataset-1 (a), and Dateset-2 (b). It can be seen that when *k*=4, our algorithm KFV archived the best performance. Note: There is one PFM with length 4 in both Dataset-1 (MA0094) and Dataset-2 (M00653), thus these two motifs were excluded for the ROC analysis for KFV with K=5 in the respective datasets.
