# Supplementary material for: A Novel Alignment-Free Method for Comparing Transcription Factor Binding Site Motifs
Source: PLoS One. 2010 Jan 20;5(1):e8797. doi: 10.1371/journal.pone.0008797 (PMC2808352; doi:10.1371/journal.pone.0008797)
Supplement: Figure S2 — The logos of 15 familial binding profiles (FBP) based on the 16 clusters from the motif tree (Figure 2). (0.21 MB DOC) [file pone.0008797.s002.doc]

**Figure S2**. **The logos of 15 familial binding profiles (FBP) based on the 16 clusters from the motif tree (Figure 2)**. The motif logo for the singleton cluster which contains only one PFM (PBX1) is not shown.


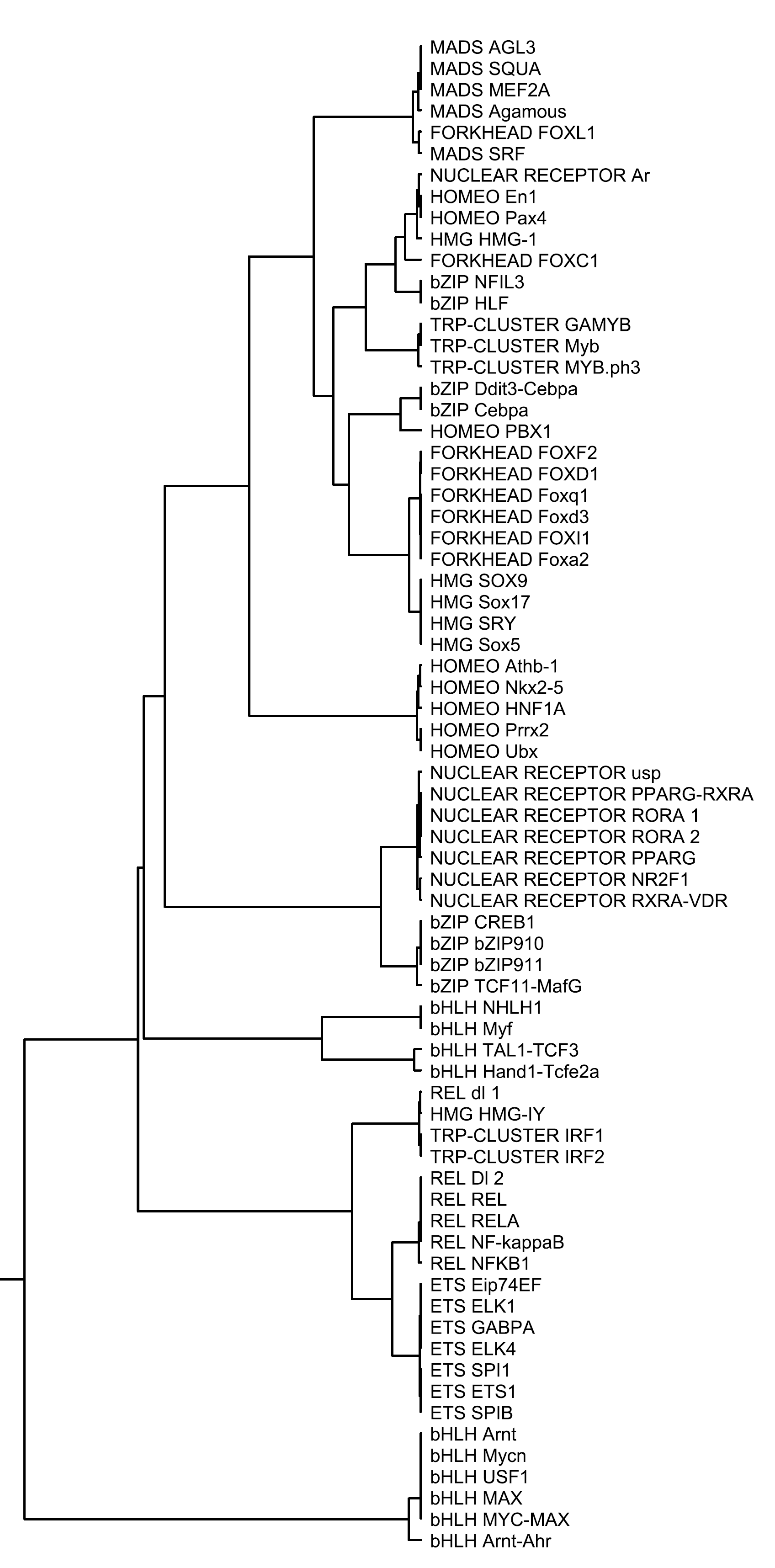

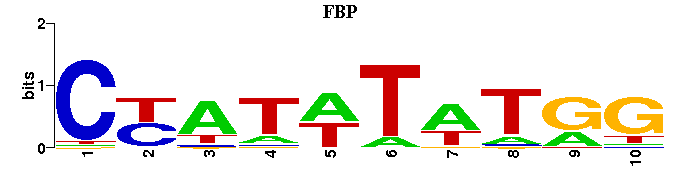

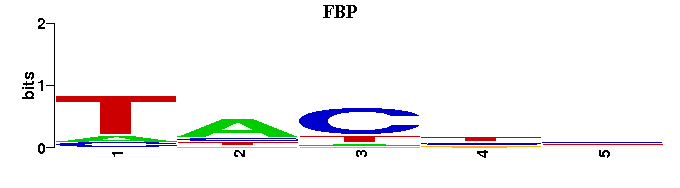

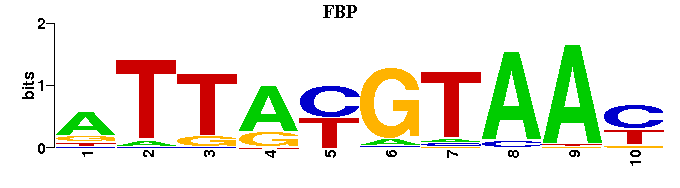

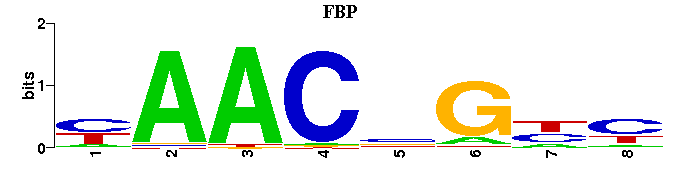

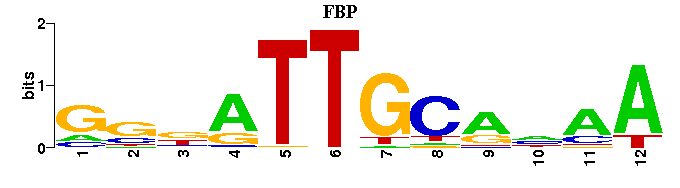

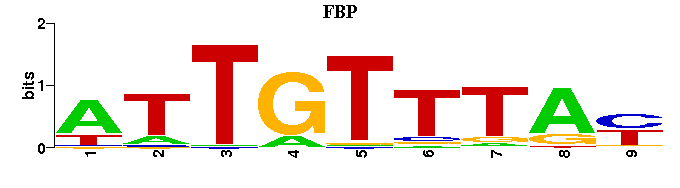

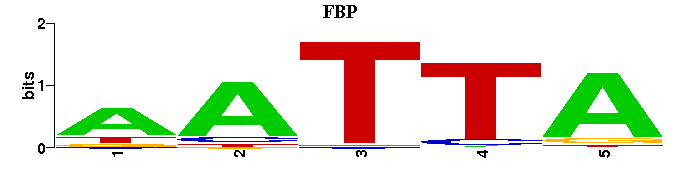

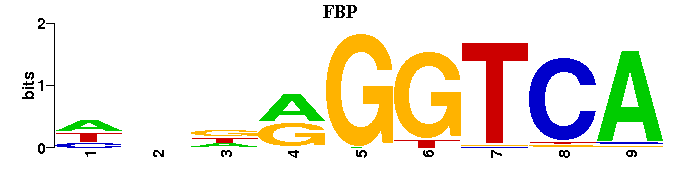

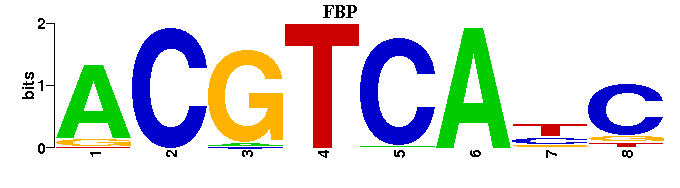

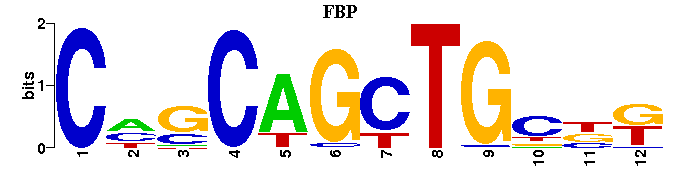

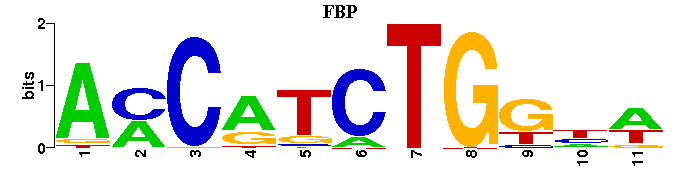

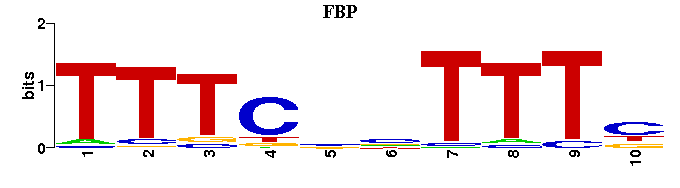

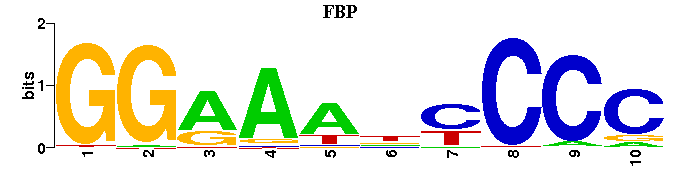

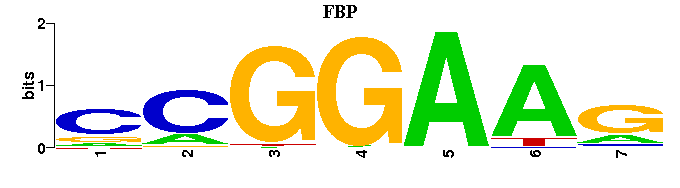

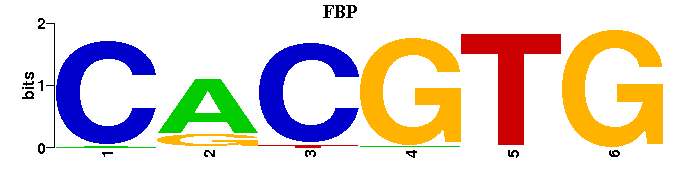


FBPs
