# Supplementary material for: A Novel Alignment-Free Method for Comparing Transcription Factor Binding Site Motifs
Source: PLoS One. 2010 Jan 20;5(1):e8797. doi: 10.1371/journal.pone.0008797 (PMC2808352; doi:10.1371/journal.pone.0008797)

**Figure S3**. **The motif tree of the 71 non-ZF PFMs in Dataset-1 constructed using MoSta.** The tree is based on the pairwise motif similarity scores calculated by MoSta (S_max_ score, 0.5 CG content and balanced threshold). Since the S_max_ scores from MoSta contain both positive and negative values, pairwise distances were calculated using the transformation: $d=\frac{1}{e^{S_{\max}}}$. Hierarchical clustering was performed using UPGMA algorithm implemented in PHYLIP.


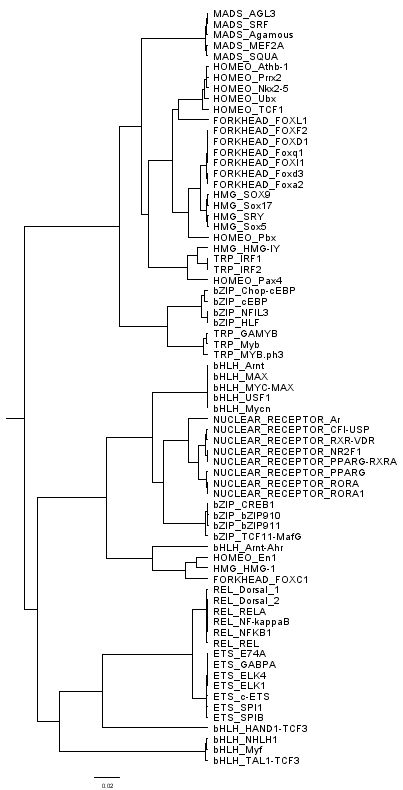

Supplement: Figure S3 — The motif tree of the 71 non-ZF PFMs in Dataset-1 constructed using MoSta. (0.04 MB DOC) [file pone.0008797.s003.doc]
