# Supplementary material for: A Novel Alignment-Free Method for Comparing Transcription Factor Binding Site Motifs
Source: PLoS One. 2010 Jan 20;5(1):e8797. doi: 10.1371/journal.pone.0008797 (PMC2808352; doi:10.1371/journal.pone.0008797)
Supplement: Figure S4 — Distributions of motif length in the three datasets. (0.03 MB DOC) [file pone.0008797.s004.doc]

**Figure S4**. **Distributions of motif length in the three datasets.**
