# Supplementary material for: A Novel Alignment-Free Method for Comparing Transcription Factor Binding Site Motifs
Source: PLoS One. 2010 Jan 20;5(1):e8797. doi: 10.1371/journal.pone.0008797 (PMC2808352; doi:10.1371/journal.pone.0008797)
Supplement: Table S1 — Comparison of the running times of KFV, STAMP, and MoSta. (0.03 MB DOC) [file pone.0008797.s005.doc]

**Table S1**. Comparison of the running times of KFV, STAMP, and MoSta

| Dataset statistics | | | | | Algorithms tested. | | | | |
| --- | --- | --- | --- | --- | --- | --- | --- | --- | --- |
| # | Source | Average length | Min length | Max length | KFV  k=3 | KFV  k=4 | KFV  k=5 | STAMP | MoSta |
| 1 | Jaspar | 10.39 | 4 | 30 | <1s | 1s | 3s | 2s | 3m38s |
| 2 | TRANSFAC | 12.14 | 4 | 29 | 2s | 4s | 16s | 6s | 24m35s |
| 3 | Jaspar | 10.6 | 4 | 22 | 2s | 7s | 21s | 1m8s | 1h49m50s |

*The tested was conducted by computing all-to-all similarity/distance scores, and speed was measured in wall clock time. All the tests were performed on an iMac desktop computer with an Intel 2.8GHz Core 2 Duo CPU and 4GB memory. Both STAMP and MoSta were written in C++ and source codes were downloaded from their respective websites and compiled on the above mentioned machine. KFV was implemented using both Perl and C++ (the procedure for creating k-mer vector dataset was written in Perl and the calculation of distances was implemented in C++. The running time in this table for KFV is the sum of the running time of the Perl script and the C++ program). Primary parameters for the three algorithms are as follows: KFV (k-mer size: k= 3, 4 and 5, vector distance: cosine angle); STAMP (column comparison: Pearson correlation coefficient, position alignment: ungapped Smith-Waterman); MoSta (GC content: 0.5, threshold-method: balanced)
